# First year update as cardiovascular perfusion’s open access international journal

**DOI:** 10.1051/ject/2024002

**Published:** 2024-03-15

**Authors:** Raymond K Wong

With this issue, JECT has completed our very first year as an open-access journal! I am happy to report that the transition has been incredibly smooth and met all the expectations I laid out a couple of years ago when we were first contemplating these modernization initiatives after ending print copies. Beyond making all our published articles free to any reader worldwide, we sorely needed a new highly functional journal website, permanent links to each article, searchable digitized archives going back to the 60s and more. Having permanent DOI (Digital Object Identifier) links, which we never had before for example, makes it so much easier for our authors and readers to share JECT articles. We will of course continue making tweaks to the website as needed.

In recent weeks, we have successfully attained COPE membership (Committee on Publication Ethics) and indexing in the DOAJ (Directory of Open Access Journals). Being indexed in the DOAJ required us to meet rigorous standards, so this is an important early accomplishment for our journal. DOAJ indexing gives us greater validation amongst the scientific community, with some research funders requiring publishing in DOAJ indexed journals only. Inclusion in DOAJ should therefore help propel us to achieve other goals going forward.

Our new website is functioning well. It is clean, attractive, and easy to navigate. All of our previous issues and articles have been migrated and there are advanced search tools available. One slick new feature that I would like to bring to your attention is the flipbook which is usually available within a week of each issue’s publication. If you missed the old days when you could flip through a hard copy of JECT, this comes very close. This is also where you are able to see the advertisements from our industry supporters who are critical in helping us keep author APCs (Article processing charges) to reasonable amounts. So, please use the flipbook as much as you can! Furthermore, we are considering the viability of printing a limited number of hardcopy versions of these beautiful flipbooks for those who would like to purchase and handle actual hardcopies. If you are one of those people, please contact us at ject@amsect.org for more information and to let us know so that we can gauge interest in this option.

For our sponsoring society, AmSECT, all these evolutions have been highly consequential. It is very difficult for relatively small societies to support a journal all by themselves. Societies such as AmSECT have limited resources and expertise to sustain a quality and competitive journal. Having new publishing partners and converting our publication model were necessary steps in order for this publication to continue and progress. EDP Sciences, operating worldwide but based in France, has facilitated, and accelerated all of our recent advances. It has been a pleasure working with their consummate professionals this past year. In fact, I would like to list at least a few individuals here because they normally work behind the scenes assisting with operations and issue production.

First is Anne Ruimy, PhD, a senior publisher based in France who was critical in setting us up during our transitional period. Next, Vijala Kiruvanayagam, PhD, our publishing editor based in the UK has been pivotal in our new COPE and DOAJ memberships and continues to shepherd our efforts to mature as a modern open-access journal. Last, but not least, is Aliénor Decours-Perez, a Journal Publisher based in France, who has done much to keep our issue productions on time and in good order. There are of course many others taking care of production, marketing, finances, and website maintenance and I wish to thank all of them for their tireless work.

In conclusion, this update reports that our transition and first year of operations as an open-access journal has been successful; and our partnership with new publishers, EDP Sciences has been productive. Together with our authors, peer reviewers, and readers, we will continue to advance JECT to new heights!

